# Central sensitization in opioid use disorder: a novel application of the American College of Rheumatology Fibromyalgia Survey Criteria

**DOI:** 10.1097/PR9.0000000000001016

**Published:** 2022-07-07

**Authors:** O. Trent Hall, Julie Teater, Kara M. Rood, K. Luan Phan, Daniel J. Clauw

**Affiliations:** Departments of a Psychiatry and Behavioral Health; b Obstetrics and Gynecology, Ohio State University Wexner Medical Center, Columbus, OH, USA; c Department of Anesthesiology, Chronic Pain and Fatigue Research Center, University of Michigan, Ann Arbor, MI, USA; d Division of Rheumatology, Department of Internal Medicine, University of Michigan, Ann Arbor, MI, USA

**Keywords:** Central nervous system sensitization, Opioid-related disorders, Fibromyalgia, Chronic pain

## Abstract

**Introduction:**

Central sensitization (CS) involves dysfunctional central nervous system pain modulation resulting in heightened pain perception. Central sensitization is not commonly assessed among patients with opioid use disorder (OUD), despite the fact that pain has been implicated in the development, maintenance, and relapse of OUD and chronic opioid use may produce opioid-induced hyperalgesia. Central sensitization is a plausibly important mechanism underlying the complex relationship between OUD and chronic pain. However, this premise is largely untested.

**Methods:**

Participants with OUD (n = 141) were recruited from an academic addiction treatment center in Columbus, Ohio. An established surrogate measure of CS, the American College of Rheumatology 2011 Fibromyalgia Survey Criteria, was administered using electronic survey. Participants also responded to questions about pain interference (Brief Pain Inventory), quality of life (RAND-36), and items regarding pain beliefs and expectations of pain and addiction treatment. Descriptive analyses, Spearman rho correlations, and Mann–Whitney *U* tests were performed.

**Results:**

Hypothesized relationships were confirmed between degree of CS, pain interference, and health-related quality of life. Degree of CS was also positively correlated with greater endorsement of pain as a reason for the onset, maintenance, and escalation of OUD; treatment delay; and OUD relapse. Participants with the American College of Rheumatology 2011 Fibromyalgia Survey Criteria ≥13 had significantly greater endorsement of pain as a reason for delaying OUD treatment, continuing and increasing opioid use, and precipitating OUD relapse.

**Conclusions:**

This study provides early evidence CS may underlie previously observed connections between clinically salient features of chronic pain and OUD, potentially informing future mechanistic research and precision treatment.

## Abstract 2

Central sensitization may be an underlying mechanism linking chronic pain and opioid use disorder associated with opioid use disorder onset, maintenance, escalation, treatment delay, and relapse.

## 1. Introduction

Central sensitization (CS), like closely related construct nociplastic pain, is believed to result from aberrant functioning of the nociceptive system leading to heightened pain perception.^36,43,100^ Central sensitization is not commonly assessed among patients with opioid use disorder (OUD), despite the fact that chronic opioid use is known to produce a neurobiological state very similar to CS, opioid-induced hyperalgesia (OIH), and pain has been implicated in the development, maintenance, and relapse of OUD.^6–8,58,93^ Indeed, chronic pain and OUD are often comorbid, frustrating treatment and compromising outcomes for both conditions.^81,84^ Although relationships between chronic pain and OUD are complex and incompletely understood, CS may be an important underlying factor warranting further study.^8,83,93^

Converging evidence points to environmental, genetic, and neurobiological overlap between CS and OUD. Early life adversity increases the risk of both CS and OUD.^44,59,70,82,88,101^ Polymorphisms of the mu-opioid receptor gene *OPRM1* may predispose individuals to CS and OUD.^22,32,34,41,80^ Neuroimaging has associated decreased gray matter volume in the insula and anterior cingulate cortex with CS and OUD.^13,43,79^ These structures are involved in affective pain processing as well as conscious urges to take drugs.^47,65,79^ Together, these findings suggest CS may be a promising precision medicine target for chronic pain and OUD.

Assessing CS in OUD might increase the understanding of comorbid chronic pain and OUD, perhaps allowing for the identification of subgroups for whom centralized or nociplastic pain presents a barrier to OUD remission. Characterizing CS in this population could also aid in the selection of more targeted OUD treatments as US Food and Drug Administration approved medications for OUD (MOUD) each have distinct pain pharmacology, and OUD behavioral counseling may be augmented with therapies proven to improve pain coping among individuals with CS.^24,37,40,85^ Therefore, a clinically applicable measure of CS in OUD would be of great importance.

A strong candidate measure is the American College of Rheumatology 2011 Fibromyalgia Survey Criteria (ACR-FMS).^97^ The American College of Rheumatology 2011 Fibromyalgia Survey Criteria has been used to assess CS among patients with other chronic pain conditions (ie, rheumatoid arthritis, postoperative pain, and low back pain).^2,5,15,31,42,51,67^ However, no previous research has examined CS using ACR-FMS in patients with OUD. Consequently, the prevalence of CS among patients with OUD is unknown, and any relationships existing between CS and clinically salient features of pain or addiction among this population remain untested.

Therefore, the present work aims to describe the use of a surrogate measure of CS among a clinical sample of individuals with OUD; test group differences based on a previously established ACR-FMS cut point; and explore potential relationships between ACR-FMS scores and pain interference, quality of life, pain-beliefs, and care expectations. We specifically probed pain-coping motivated opioid use, awareness of the opioid hyperalgesia phenomenon, and pain-based negative expectations of medical care and addiction treatment.

## 2. Methods

Before initiation, the study protocol was approved by the Ohio State University Wexner Medical Center Institutional Review Board. Survey data were collected on tablet computers using REDCap, a web platform capable of securely collecting personal health information and managing online databases. Participants provided verbal consent and were monetarily compensated for their time.

### 2.1. Study sample

Study inclusion criteria were adults with OUD defined by the presence of at least 2 of 11 symptoms of OUD during the past 12 months as described in the Diagnostic and Statistical Manual of Mental Disorders, Fifth Edition (DSM-5).^1^ Exclusion criteria were inability to provide informed consent, read, or comprehend survey items. There were no other inclusion or exclusion criteria. Potential participants were not prescreened for the presence of pain as an inclusion or exclusion criterion.

One-hundred forty-four individuals were consecutively recruited between July 7, 2021, and December 10, 2021, from the patient pool at Ohio State University Wexner Medical Center Addiction Care at Talbot Hall. This facility provides OUD treatment including partial hospitalization, intensive outpatient, group and individual counseling, medically supervised withdrawal, and medication management (buprenorphine and naltrexone). Recruitment was conducted by trained addiction treatment professionals during the course of routine evaluation. Participants accessed the questionnaire on a tablet computer in a private examination room and were not allowed to interact with one another while completing the survey.

Eight individuals were offered participation but declined. One case was excluded for violating inclusion criteria. Two cases were excluded for noncompletion of the primary measure of interest (ACR-FMS). Other cases were missing health-related quality of life (5, 3.7%) or demographics (10, 7.1%). These were retained in the final sample but excluded from specific correlational analyses where necessary, as noted. The final sample was 141 (n = 141).

### 2.2. Measures

The survey included DSM-5 OUD criteria (as assessed by an addiction provider on the date of participation), a demographic questionnaire (sex, race, ethnicity, employment status, income, education, housing status, and substance use treatment needs), as well as the following validated instruments and original items. Age was unintentionally omitted from the survey.

### 2.3. 2011 Fibromyalgia Survey Criteria

Participants' burden of pain and related symptoms was assessed by the ACR-FMS.^44,96,97^ Although it has principally been used for fibromyalgia diagnosis, ACR-FMS has also been purposed as a measure of CS and related construct nociplastic pain. Although both CS and nociplastic pain may be assessed by ACR-FMS, the definition of these constructs bears further distinction. CS is “*Increased responsiveness of nociceptive neurons in the central nervous system to their normal or subthreshold afferent input,”* while nociplastic pain is defined as *“Pain that arises from altered nociception despite no clear evidence of actual or threatened tissue damage causing the activation of peripheral nociceptors or evidence for disease or lesion of the somatosensory system causing the pain.”*^49^ The American College of Rheumatology 2011 Fibromyalgia Survey Criteria characterizes pain location by body region (0–19) as well as the severity of associated symptoms including problems thinking, sleep difficulty, and fatigue (0–12). As a continuous measure (range 0–31), ACR-FMS has been used as a proxy for the degree of CS or as an indicator of probable fibromyalgia (ACR-FMS ≥ 13, sensitivity 96.6% and specificity 91.8%).^97^ The American College of Rheumatology 2011 Fibromyalgia Survey Criteria have previously quantified CS and have been found to be robustly predictive of pain, disability, and treatment outcomes in diverse clinical populations.^2,5,11,12,15,31,51,66,67,94,95,98,99^ The American College of Rheumatology 2011 Fibromyalgia Survey Criteria can also be used to assess how much nociplastic pain contributes to the symptom burden in other chronic pain conditions.^36^

### 2.4. Pain interference

The degree to which pain interfered with participants' daily experience was measured with the pain interference subscale of the Brief Pain Inventory (BPI).^16^ This subscale assesses pain interference across two dimensions including “affective interference” (sleep, relationships, life enjoyment, and mood) and “activity interference” (general activity, normal work, and walking).^17^ Items comprising the BPI pain interference subscale are scored 0 “*Does not interfere”* to 10 *“Completely interferes”* and may be summed together (range 0–70). Alternatively, the “affective interference” and “activity interference” dimensions may be treated as separate subscales with ranges 0 to 40 and 0 to 30, respectively.^17^ It has been proposed that treating “affective interference” and “activity interference” as separate subscales is appropriate because each suggests a different target for intervention and either may be unequally affected potentially negating the value of their sum as an index of pain interference.^63^

### 2.5. Health-related quality of life

Health-related quality of life was assessed by the Research and Development (RAND) Corporation RAND 36-Item Health Survey 1.0 (RAND-36).^45^ RAND-36, also referred to as the Short Form 36 (SF-36), is a widely used comprehensive survey measuring health along 8 domains: general health, physical functioning, mental health, social functioning, vitality, bodily pain, role limitations due to physical health, and role limitations due to emotional problems. Scoring RAND-36 involves transforming each of its 36 items linearly to a range of 0 to 100 and averaging items by domain.^21,45^ Higher domain scores represent greater health-related quality of life. The psychometric properties of RAND-36 have been extensively studied confirming its validity and reliability.^9,21,64,86,87^

### 2.6. Original items regarding pain-related beliefs and care expectations

Eight original items were created to measure beliefs and expectations regarding pain and OUD treatment which might reflect opioid use motivations or relate to clinical outcomes for individuals with pain and OUD. Responses were scaled as *strongly disagree (1), disagree (2), neutral (3), agree (4),* or *strongly agree (5).* Table 1 presents these original items.

**Table 1:** Original items regarding pain beliefs and care expectations.

Pain Beliefs
I first started using opioids because I was in pain.
Pain is a major reason why I have kept using opioids.
I find myself needing more and more opioids to control my pain.
It is possible for opioids to make pain worse over time.
Care Expectations
Once doctors know you have an addiction, they would not help you with your pain.
I have put off going to the doctor for my pain because I do not want treated like a “drug addict.”
I have put off getting treatment for opioid use disorder because I am afraid my pain will be worse when I stop using opioids.
I am worried pain will cause me to relapse in the future.

Responses were scaled as *strongly disagree (1), disagree (2), neutral (3), agree (4),* or *strongly agree (5).*

### 2.7. Data analysis

Descriptive analyses providing measures of central tendency, frequency, and percentages were conducted to characterize demographic features as well as OUD severity, CS, pain interference, health-related quality of life, pain-related beliefs, and care expectations. This was followed by a series of Spearman correlations to probe hypothesized associations between CS (ACR-FMS) and other variables of interest. For Spearman correlations, the size of r_s_ was interpreted 0.1 = small, 0.3 = medium, and 0.5 = large.^18^ Mann–Whitney U tests were run to determine differences in pain beliefs and care expectations between participants above and below the ACR-FMS ≥ 13 cut point. All tests were 2-tailed and were deemed significant at α < 0.01. Statistical analyses were performed using SPSS software (Version 27.0, SPSS. Inc).

## 3. Results

### 3.1. Sample characteristics

Demographic information was provided by 131 (92.9%) participants. Of those who completed the demographic questionnaire, 53 (40.5%) were female and 78 (59.5%) were male. Thirty-four reported their race as Black (26%), 94 White (71.8%), and 3 any other race (2.3%). Most (126, 96.2%) claimed non-Hispanic ethnicity. Age was unintentionally omitted from the questionnaire, but participants were adults between age 18 and 88 years. All 141 participants responded to a multiple answer question inquiring about the types of substances for which they were seeking treatment. Allowed to choose more than one substance, most reported they were seeking addiction treatment for fentanyl (107, 75.9%), followed by heroin (47, 33.3%) and prescription opioids (29, 20.6%). Little variance was observed in OUD severity (mean number of OUD criteria present = 10.60 ± 1.34). Most (137, 97.2%) met criteria for severe OUD (6 or more DSM-5 OUD criteria). Three (2.1%) participants had moderate OUD (4–5 DSM-5 OUD criteria), and 1 (0.7%) had mild OUD (2–3 criteria). Table 2 presents sample characteristics.

**Table 2:** Sample characteristics.

Characteristic	Participants
Racial identity, n (%)	
Black	34 (26.0)
White	94 (71.8)
Any other race	3 (2.3)
Ethnicity, n (%)	
Hispanic	5 (3.8)
Non-Hispanic	126 (96.2)
Sex, n (%)	
Female	53 (40.5)
Male	78 (59.5)
Substance use, n (%)	
Fentanyl	107 (75.9)
Heroin	47 (33.3)
Prescription opioid	29 (20.6)
Annual income, n (%)	
Less than $20,000	88 (67.2)
$20,000–$34,999	23 (17.6)
$35,000–$49,999	15 (11.5)
$50,000–$74,999	5 (3.8)
$75,000 or greater	0 (0)
Employment, n (%)	
Unable to work	20 (15.3)
Working	34 (26.0)
Not working	77 (58.8)
Education, n (%)	
Less than high school	37 (28.2)
High school or GED	45 (34.4)
Some postsecondary	27 (20.6)
Postsecondary degree	22 (16.8)
Stable housing, n (%)	
Yes	93 (71.0)
No	38 (29.0)

Percentages for substance use based on n = 141. All other percentages based on n = 131 due to 10 participants with missing demographics. Age unintentionally omitted.

### 3.2. Central sensitization (the American College of Rheumatology 2011 Fibromyalgia Survey Criteria)

**Figure 1.**
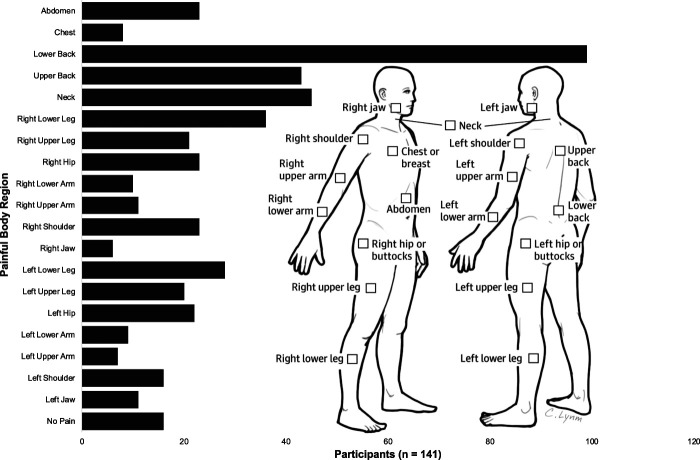
Frequency of self-reported pain by body region. Anatomic illustration from the ACR-FMS/Michigan body map.^14^ ACR-FMS, American College of Rheumatology 2011 Fibromyalgia Survey Criteria.

Pain was prevalent in this clinical sample of participants with OUD. The sample mean, minimum, and maximum ACR-FMS scores were 9.88 ± 4.91, 0 and 23, respectively. Total scores were normally distributed per Shapiro–Wilk (*P* > 0.05). One hundred twenty-five participants (88.7%) reported at least 1 painful body region. Widespread pain was common, with participants averaging 3.27 ± 2.83 painful body regions. Pain was most often reported in the low back (99, 70.2%), neck (45, 31.9%), and upper back (43, 30.5%). Figure 1 shows the frequency of pain by body region.

The number of participants reporting moderate or severe cognitive symptoms, fatigue, or waking unrefreshed was 62 (44.0%), 87 (61.7%) and 93 (66%), respectively. Among participants with any pain, the scores of 39 (31.2%) were consistent with the diagnosis of fibromyalgia (ACR-FMS ≥ 13).

### 3.3. Pain interference (Brief Pain Inventory) and health-related quality of life (RAND-36)

Pain interference was common among participants, differentially affecting affective and activity domains. The BPI pain interference subscale total, affective interference, and activity interference dimensions were not normally distributed as assessed by Shapiro–Wilk tests (*P* < 0.05). Median scores were 39 (IQR = 26–50) of 70 for the BPI pain interference subscale, 24 (IQR = 15–32) of 40 for the affective interference dimension, and 14 (IQR = 6–20) of 30 for the activity interference dimension.

Responses to RAND-36 1.0 indicated overall decreased health-related quality of life among the sample. Shapiro–Wilk tests of normality showed none of the 8 life domains of RAND-36 were normally distributed (*P* < 0.05). Median health-related quality of life was 50 or lower (100 denotes optimal life quality) in 7 of 8 life domains: general health 45 (IQR = 35–60), mental health 48 (IQR = 36–59), social functioning 50 (IQR = 25–63), vitality 40 (IQR = 30–50), bodily pain 45 (IQR = 33–68), role limitations due to physical health 50 (IQR = 3–100), and role limitations due to emotional problems 0 (IQR = 0–67). Physical functioning was the exception with a sample median of 75 (IQR = 45–90).

### 3.4. Pain-related beliefs and care expectations

Pain-related beliefs including endorsement of pain-related onset, maintenance and escalation of OUD, as well as awareness of the OIH phenomenon were noted. Eighty-six of 141 participants (61.0%) agreed or strongly agreed that they first used opioids *“because I was in pain.”* Seventy-seven (54.6%) participants indicated that pain played an important role in maintaining their addiction by agreeing or strongly agreeing pain was *“a major reason”* they continued using opioids. Eighty-four (59.6%) affirmed that pain-coping motivated their escalating opioid use *“I find myself needing more and more opioids to control my pain.”* Awareness of OIH was expressed by 68 (48.2%) participants who affirmed *“It is possible for opioids to make pain worse over time.”*

A subset of participants held negative expectations of both pain and addiction treatment. Physician stigma against patients with OUD was perceived as a barrier to pain treatment by 78 (55.3%) participants who agreed or strongly agreed doctors withhold pain treatment from patients with an addiction. Eighty-four (59.6%) participants endorsed delaying needed pain treatment for fear of being stigmatized for their OUD. Seventy-six (54.0%) indicated that they had delayed accessing OUD treatment because they worried their pain would be worse in recovery from OUD. Finally, pain-triggered OUD relapse was anticipated by 81 (57.4%) participants whom agreed or strongly agreed *“I am worried pain will cause me to relapse in the future.”*

### 3.5. Correlational analyses

Correlational analyses revealed relationships between CS, pain interference, and health-related quality of life among this clinical sample of participants with OUD. Central sensitization was significantly associated with total BPI pain interference (r_s_ (141) = 0.539, *P* < 0.001) as well as its affective interference (r_s_ (141) = 0.559, *P* < 0.001) and activity interference domains (r_s_ (141) = 0.457, *P* < 0.001). Significant negative associations were also noted between CS and 7 of 8 domains of health-related quality of life including general health (r_s_ (136) = −0.580, *P* < 0.001), mental health (r_s_ (136) = −0.547, *P* < 0.001), social functioning (r_s_ (136) = −0.592, *P* < 0.001), physical functioning (r_s_ (136) = −0.420, *P* < 0.001), vitality (r_s_ (136) = −0.480, *P* < 0.001), bodily pain (r_s_ (136) = −0.632, *P* < 0.001), and role limitations due to emotional problems (r_s_ (136) = −0.474, *P* < 0.001). No significant association was found between CS and role limitations due to physical problems.

**Figure 2.**
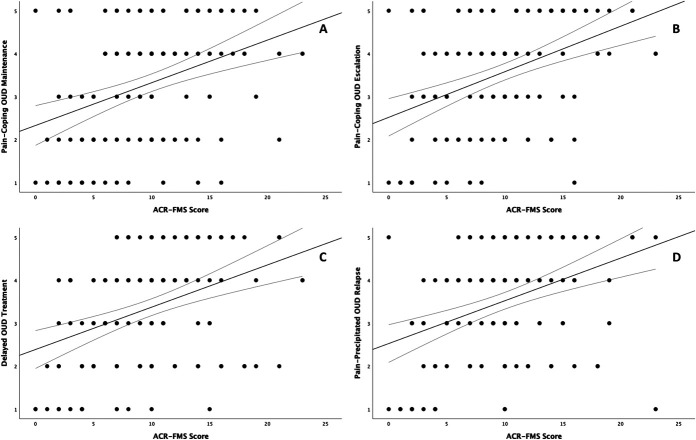
Relationships between total ACR-FMS score and endorsement of A. pain as a major reason for continuing opioid use, B. needing more and more opioids to control pain, C. delaying OUD treatment for fear that pain will be worse when stopping opioids, and D. worry about pain-precipitated OUD relapse. Responses (Y axis) to original questions were on a 5-point Likert scale and scaled as *strongly disagree (1), disagree (2), neutral (3), agree (4),* or *strongly agree (5).* ACR-FMS, American College of Rheumatology 2011 Fibromyalgia Survey Criteria; OUD, opioid use disorder.

Central sensitization was also correlated with pain-related beliefs and expectations of pain and addiction treatment. Small, but significant associations were found between CS and pain-related onset of OUD (r_s_ (141) = 0.273, *P* = 0.001), awareness of OIH (r_s_ (141) = 0.235, *P* = 0.005), and OUD stigma-related delay of treatment for pain (r_s_ (141) = 0.271, *P* = 0.001). Interestingly, despite its observed relationship with OUD stigma-related pain treatment delay, CS was not significantly correlated with the perception that physicians withhold pain treatment from patients with addiction. There was a modest association between CS and reporting pain was *“a major reason”* for continued opioid use (r_s_ (141) = 0.364, *P* < 0.001). Similarly sized correlations were noted between CS and pain-coping motivated escalation of opioid use (r_s_ (141) = 0.394, *P* < 0.001), delayed OUD treatment due to fear of pain exacerbation (r_s_ (141) = 0.390, *P* < 0.001), and worry about future pain-precipitated OUD relapse (r_s_ (141) = 0.395, *P* < 0.001). Figure 2 shows negative expectations of addiction treatment by ACR-FMS total score.

### 3.6. Group differences in American College of Rheumatology 2011 Fibromyalgia Survey Criteria ≥ 13 vs American College of Rheumatology 2011 Fibromyalgia Survey Criteria < 13

Group differences in health-related quality of life, pain interference, pain-beliefs, and care expectations by ACR-FMS cut point ≥13 were assessed using Mann–Whitney U tests. Distributions of all dependent variables were similar on visual inspection. Significant group differences were observed in pain interference and health-related quality of life (Table 3).

**Table 3:** Group differences in health-related quality of life and pain interference by the American College of Rheumatology 2011 Fibromyalgia Survey Criteria cut point ≥13.

Survey	ACR-FMS ≥ 13 median score	ACR-FMS < 13 median score	U	Z	*P*
BPI interference					
Total	52.00	33.00	867.0	−5.175	<0.001
Affective Domain	32.00	22.00	819.5	−5.397	<0.001
Activity Domain	19.00	12.50	1080.5	−4.194	<0.001
RAND-36					
General health	42.50	50.00	1029.5	−4.055	<0.001
Mental health	36.00	52.00	1073.0	−3.836	<0.001
Social functioning	37.50	50.00	1086.0	−3.801	<0.001
Vitality	35.00	45.00	1116.5	−3.836	<0.001
Bodily pain	27.50	57.50	715.0	−5.957	<0.001
Physical functioning	50.00	80.00	1158.0	−3.43	0.001
Emotional role limitations	0.00	0.00	1226.5	−3.48	0.001
Physical role limitations	50.00	50.75	1226.5	−1.154	0.248

All comparisons with Mann–Whitney U tests.

ACR-FMS, American College of Rheumatology Fibromyalgia Survey; BPI, Brief Pain Inventory; RAND-36, RAND 36-Item Health Survey 1.0 (a survey of health-related quality of life). Higher scores on BPI indicate greater pain interference. Lower scores on RAND-36 indicate lower quality of life.

**Figure 3.**
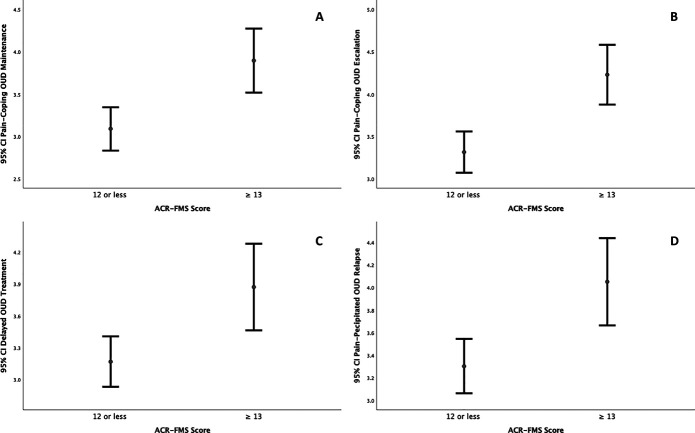
Group differences (mean with 95% confidence interval) in endorsement of A. pain as a major reason for continuing opioid use, B. needing more and more opioids to control pain, C. delaying OUD treatment for fear that pain will be worse when stopping opioids, and D. worry about pain-precipitated OUD relapse by ACR-FMS cut point ≥13 (consistent with fibromyalgia). Responses (Y axis) to original questions were on a 5-point Likert scale and scaled as *strongly disagree (1), disagree (2), neutral (3), agree (4),* or *strongly agree (5).* ACR-FMS, American College of Rheumatology 2011 Fibromyalgia Survey Criteria; OUD, opioid use disorder.

Participants with ACR-FMS ≥ 13 also differed significantly from those with ACR-FMS < 13 on self-report of pain as *“a major reason”* for continued opioid use (ACR-FMS ≥ 13 *Mdn* = 4, ACR-FMS < 13 *Mdn* = 3), U = 1287.5, z = −3.337, *P* = 0.001, pain-coping motivated escalation of opioid use (ACR-FMS ≥ 13 *Mdn* = 5, ACR-FMS < 13 *Mdn* = 4), U = 1114.0, z = −4.165, *P* = 3.1 × 10^−5^, delayed OUD treatment due to fear of pain exacerbation (ACR-FMS ≥ 13 *Mdn* = 4, ACR-FMS < 13 *Mdn* = 3), U = 1321.5, z = −3.172, *P* = 0.002, and worry about future pain-precipitated OUD relapse (ACR-FMS ≥ 13 *Mdn* = 5, ACR-FMS < 13 *Mdn* = 4), U = 1269.5, z = −3.421, *P* = 0.001. Figure 3 illustrates group differences (mean with 95% confidence interval), by ACR-FMS cut point ≥13, in endorsement of pain as a major reason for continuing opioid use, needing more and more opioids to control pain, delaying OUD treatment for fear that pain will be worse when stopping opioids, and worry about pain-precipitated OUD relapse.

## 4. Discussion

This is the first study to describe ACR-FMS as a surrogate measure of CS, or nociplastic pain, in OUD. Participants were not selected on the basis of pain. Despite this, pain and CS were prevalent. Most participants reported pain in at least one body region. Widespread pain affecting multiple body regions and symptoms associated with CS (ie, fatigue, cognitive symptoms, and abdominal cramping) were common.

Relationships of potential clinical importance were observed between CS and lived experiences of pain and OUD. Greater CS was correlated with increased pain interference, perhaps indicating nociplastic pain may be more disruptive than pain that is primarily nociceptive or neuropathic to individuals with comorbid chronic pain and OUD. Similarly, greater CS was correlated with reduced quality of life, suggesting nociplastic pain may be an important, previously unappreciated, contributor to the overall disease burden of OUD.

Central sensitization was associated with endorsement of pain-related onset, maintenance and escalation of OUD, as well as awareness of OIH. That CS was associated with endorsement of pain-related OUD onset suggests nociplastic pain should be investigated as an independent risk factor for OUD, distinct from pain that is primarily nociceptive or neuropathic. Similarly, the present findings might indicate CS has a role in OUD disease progression. Nociplastic pain might serve as an aversive condition akin to the discomfort of withdrawal in well-established antireward systems and negative reinforcement processes central to the brain disease model of addiction.^38,51–53,89^ Intriguingly, although almost 90% of the sample reported pain, less than half endorsed awareness of OIH. This awareness was positively correlated with CS. Aligning with previous research implicating CS in OIH, this finding suggests the utility of ACR-FMS in future studies of CS and OIH.^60,76^

Central sensitization was associated with negative expectations of pain treatment. Central sensitization was correlated with delaying pain treatment out of concern for OUD stigma. OUD stigma is a barrier to pain treatment.^57^ Anticipation of OUD stigma involves expecting that one's pain complaints will not be believed.^26^ This may be particularly relevant for patients with nociplastic pain. However, CS was not associated with the belief that doctors withhold pain treatment from patients with OUD, suggesting opportunity for engagement.

Central sensitization also related to negative expectations of addiction treatment. Fear of pain exacerbation on stopping opioids as a reason for delaying OUD treatment was associated with CS. The effects of opioid cessation on pain levels among individuals with comorbid nociplastic pain and OUD are unknown. However, opioid tapering has improved pain among patients with fibromyalgia.^23,35,46^ Patients should be counseled about pain in OUD recovery and should have access to evidence-based pain treatment. Finally, CS was robustly associated with fear of pain-precipitated OUD relapse. Individuals with comorbid chronic pain and OUD are 3 to 5 times more likely to relapse than those with OUD alone.^58^ We speculate that CS may potentially mediate the increased risk of OUD relapse conferred by chronic pain, although additional research is needed.

Nearly one-third of the sample exhibited ACR-FMS scores consistent with fibromyalgia (ACR-FMS ≥ 13); a remarkably high proportion given the population prevalence of fibromyalgia is estimated to be between 2% and 4%.^36^ These individuals reported greater pain interference and lower quality of life, again suggesting nociplastic pain as an important contributor to OUD disease burden. In addition, participants with ACR-FMS ≥ 13 were more likely to endorse pain as a reason for continuing and escalating their opioid use and for delaying OUD treatment. The ACR-FMS ≥ 13 group also exhibited significantly greater fear of pain-triggered OUD relapse. Although further research is needed, we speculate that ACR-FMS ≥ 13 may be a clinically useful delineation indicating increased risk of poor treatment outcomes among patients with comorbid chronic pain and OUD.

Existing research aids interpretation of the present work. Hyperalgesia and decreased pain tolerance have been identified among individuals with OUD.^30^ These findings have been replicated among patients before starting MOUD (methadone and buprenorphine), during MOUD treatment, and in OUD remission.^3,19,29,74^ Similar experiments have related hyperalgesia and decreased pain tolerance to CS among individuals with fibromyalgia.^28,69,72,90,91,92^ Taken together, these findings support our notion that CS may be an important mechanism underlying comorbid chronic pain and OUD.

Once controversial, brain mechanisms linking OUD and chronic pain are now supported by substantial literature.^33,77^ A recent review article detailed common brain mechanisms of chronic pain and addiction including reward deficiency, impaired inhibitory control, incentive sensitization, aberrant learning, and antireward allostatic neuroadaptations. This study contributes to this literature by demonstrating ACR-FMS as a tool for studying CS in relation to antireward allostatic neuroadaptations deployable in both the clinical and laboratory settings.

Beyond basic neuroscience, other genetic and behavioral research links CS and OUD. *OPRM1* polymorphisms may predispose individuals to CS and OUD.^22,32,34,41,80^ Childhood adversity is a risk factor for nociplastic pain as well as prescription opioid misuse, OUD onset, relapse, and opioid overdose.^27,48,62,70,71,82^ Traumatic stress leads to CS and increased drug intake in animal models and is associated with elevated clinical markers of CS and drug use in humans.^4,44,54,61,75^ Furthermore, posttraumatic stress disorder and fibromyalgia have additive effects on OUD risk.^6^ Given these substantially overlapping susceptibilities, we posit that CS may be a potent precision medicine target for comorbid chronic pain and OUD that is easily measurable (by ACR-FMS) in clinical populations.

The present work has many implications. ACR-FMS may be useful in increasing the understanding of comorbid chronic pain and OUD. Previous studies of comorbid chronic pain and OUD have not benefitted from recent advancements in the mechanistic taxonomy of pain. In 2016, the International Association for the Study of Pain (IASP) added nociplastic as a third mechanism of pain, in addition to existing categories nociceptive and neuropathic.^36^ Mechanistic studies of comorbid chronic pain and OUD are urgently needed. ACR-FMS may aid in the detection of CS in OUD, potentially facilitating the identification of subgroups for whom centralized or nociplastic pain presents a barrier to OUD remission. Longitudinal studies using ACR-FMS are necessary to determine the role of CS in the onset, maintenance, escalation, and relapse of OUD.

The American College of Rheumatology 2011 Fibromyalgia Survey Criteria might also improve the precision of treatments for comorbid chronic pain and OUD by allowing randomized trials to control for primary underlying pain mechanism (nociplastic vs neuropathic or nociceptive). Currently approved MOUD agents each have distinct pain pharmacology with plausibly differential effectiveness based on primary underlying pain mechanism, although their effect on CS in OUD treatment populations is unclear. In addition to agonizing µ-opioid receptors, methadone inhibits serotonin and norepinephrine reuptake and antagonizes N-methyl-d-aspartate receptors. These secondary actions have theoretical benefit in modulating nociceptive stimuli propagation and inhibiting the wind-up phenomenon.^56^ Buprenorphine, a partial µ-opioid agonist, has been observed to reduce CS by uncertain means.^25,55^ Temporary blockade of µ-opioid receptors by naltrexone may produce upregulation of β-endorphin and related opioid peptides as well as opioid receptors, potentially enhancing endogenous analgesia.^85^ Existing nonopioid analgesics may have variable utility for comorbid chronic pain and OUD based on the degree of CS present.^20,39,68,102^ Finally, ACR-FMS might identify patients with OUD for whom OUD behavioral counseling should be combined with psychological therapies for nociplastic pain.^24,36,68^ Such counseling should include pain physiology education, promotion of physical activity, sleep hygiene, stress reduction, and improved diet.^36,78^

### 4.1. Study strengths and limitations

A relatively large clinical sample and use of validated measures with low levels of missing data were notable strengths of the present work. Our study was limited by its cross-sectional design which prevented testing of relationships between CS and clinical outcomes. Other limitations involve unclear generalizability. Although we did not recruit participants based on the presence of pain, we also did not collect data regarding pain diagnoses or pain treatments. We did not control for the type (ie, buprenorphine, naltrexone, and level of behavioral care) or duration of OUD treatment or for psychiatric comorbidities. Most had severe OUD and therefore may not represent populations with mild or moderate OUD. Future prospective cohort studies should examine baseline ACR-FMS and serial ACR-FMS scores in relation to measurable aspects of treatment for comorbid chronic pain and OUD.

## 5. Conclusion

This study provides new mechanistic insight into the relationship between OUD and chronic pain. We described the use of ACR-FMS as a surrogate measure for CS in a clinical sample with OUD. Although participants were not selected based on the presence of pain, nociplastic pain was prevalent in the sample. Hypothesized correlations were confirmed between degree of CS, pain interference, and health-related quality of life. Central sensitization was also correlated with reporting pain as a reason for delaying OUD treatment and for the onset, maintenance, escalation, and relapse of OUD. Participants with ACR-FMS ≥ 13 had significantly greater endorsement of pain as a reason for delaying OUD treatment, continuing and increasing opioid use, and precipitating OUD relapse suggesting the possible utility of this cut score as a risk-indicator of poor treatment outcomes. Central sensitization may be an important underlying factor complicating the treatment of comorbid chronic pain and OUD.

## Disclosures

D.J. Clauw has testified in state lawsuits against opioid manufacturers for their role in the opioid overdose crisis. The remaining authors have no conflicts of interest to declare.

Funding was provided by the Care Innovation and Community Improvement Plan (CICIP), a program of the Ohio Department of Medicaid. The views expressed in this publication do not necessarily reflect the official policies of the Ohio Department of Medicaid nor does mention of trade names, commercial practices, or organizations imply endorsement by the government of Ohio.

## References

[1] American Psychiatric Association. Diagnostic and statistical manual of mental disorders: DSM-5: Washington: American Psychiatric Association, 2013.

[2] Aoyagi K He J Nicol AL Clauw DJ Kluding PM Jernigan S Sharma NK. A subgroup of chronic low back pain patients with CENTRAL SENSITIZATION. Clin J Pain 2019;35:869–79. 31408011 10.1097/AJP.0000000000000755 PMC7197191

[3] Athanasos P Smith CS White JM Somogyi AA Bochner F Ling W. Methadone maintenance patients are cross-tolerant to the antinociceptive effects of very high plasma morphine concentrations. PAIN 2006;120:267–75. 16427197 10.1016/j.pain.2005.11.005

[4] Back SE Gros DF Price M LaRowe S Flanagan J Brady KT Davis C Jaconis M McCauley JL. Laboratory-induced stress and craving among individuals with prescription opioid dependence. Drug Alcohol Depen 2015;155:60–7. 10.1016/j.drugalcdep.2015.08.019 PMC4582004 26342626

[5] Basu N Kaplan CM Ichesco E Larkin T Harris RE Murray A Waiter G Clauw DJ. Neurobiologic features of fibromyalgia are also present among rheumatoid arthritis patients. Arthritis Rheumatol 2018;70:1000–7. 29439291 10.1002/art.40451

[6] Bilevicius E Sommer JL Asmundson GJG El-Gabalawy R. Posttraumatic stress disorder and chronic pain are associated with opioid use disorder: results from a 2012-2013 American nationally representative survey. Drug Alcohol Depen 2018;188:119–25. 10.1016/j.drugalcdep.2018.04.005 29775955

[7] Blanco C Wall MM Okuda M Wang S Iza M Olfson M. Pain as a predictor of opioid use disorder in a nationally representative sample. Am J Psychiatry 2016;173:1189–95. 27444794 10.1176/appi.ajp.2016.15091179

[8] Boscarino JA Rukstalis MR Hoffman SN Han JJ Erlich PM Ross S Gerhard GS Stewart WF. Prevalence of prescription opioid-use disorder among chronic pain patients: comparison of the DSM-5 vs. DSM-4 diagnostic criteria. J Addict Dis 2011;30:185–94. 21745041 10.1080/10550887.2011.581961

[9] Brazier JE Harper R Jones N O'cathain A Thomas K Usherwood T Westlake L. Validating the SF-36 health survey questionnaire: new outcome measure for primary care. BMJ Br Med J 1992;305:160–4. 1285753 10.1136/bmj.305.6846.160 PMC1883187

[10] Brummett CM Hassett AL Brummett KA Clauw DJ Williams DA. The Michigan body map and its use in assessing the American College of Rheumatology Survey criteria for fibromyalgia. Arthritis Rheumatism 2011;63:S368–S.

[11] Brummett CM Janda AM Schueller CM Tsodikov A Morris M Williams DA Clauw DJ. Survey criteria for fibromyalgia independently predict increased postoperative opioid consumption after lower-extremity joint arthroplasty: a prospective, observational cohort study. Anesthesiology 2013;119:1434–43. 24343289 10.1097/ALN.0b013e3182a8eb1f PMC3867739

[12] Brummett CM Urquhart AG Hassett AL Tsodikov A Hallstrom BR Wood NI Williams DA Clauw DJ. Characteristics of fibromyalgia independently predict poorer long‐term analgesic outcomes following total knee and hip arthroplasty. Arthritis Rheumatol 2015;67:1386–94. 25772388 10.1002/art.39051 PMC4414825

[13] Cagnie B Coppieters I Denecker S Six J Danneels L Meeus M. CENTRAL SENSITIZATION in fibromyalgia? A systematic review on structural and functional brain MRI. Semin Arthritis Rheu 2014;44:68–75. 10.1016/j.semarthrit.2014.01.001 24508406

[14] Clauw DJ. Fibromyalgia: a clinical review. JAMA 2014;311:1547–55. 24737367 10.1001/jama.2014.3266

[15] Clauw DJ Hassett AL. The role of centralised pain in osteoarthritis. Clin Exp Rheumatol 2017;35(suppl 107):79–84. 28967359

[16] Cleeland C Ryan K. Pain assessment: global use of the Brief pain inventory. Ann Acad Med Singap 1994;23:129–38. 8080219

[17] Cleeland CS Nakamura Y Mendoza TR Edwards KR Douglas J Serlin RC. Dimensions of the impact of cancer pain in a four country sample: new information from multidimensional scaling. PAIN 1996;67:267–73. 8951920 10.1016/0304-3959(96)03131-4

[18] Cohen J. Statistical Power Analysis for the Behavioral Sciences (2nd ed.). Routledge, 1988. 10.4324/9780203771587

[19] Compton P Canamar CP Hillhouse M Ling W. Hyperalgesia in heroin dependent patients and the effects of opioid substitution therapy. J Pain 2012;13:401–9. 22424799 10.1016/j.jpain.2012.01.001 PMC3334366

[20] Compton P Kehoe P Sinha K Torrington MA Ling W. Gabapentin improves cold-pressor pain responses in methadone-maintained patients. Drug Alcohol Depen 2010;109:213–19. 10.1016/j.drugalcdep.2010.01.006 PMC2875370 20163921

[21] Coons SJ Alabdulmohsin SA Draugalis JR Hays RD. Reliability of an Arabic version of the RAND-36 Health Survey and its equivalence to the US-English version. Med Care 1998:428–32. 9520966 10.1097/00005650-199803000-00018

[22] Crist RC Reiner BC Berrettini WH. A review of opioid addiction genetics. Curr Opin Psychol 2019;27:31–5. 30118972 10.1016/j.copsyc.2018.07.014 PMC6368898

[23] Cunningham JL Evans MM King SM Gehin JM Loukianova LL. Opioid tapering in fibromyalgia patients: experience from an interdisciplinary pain rehabilitation program. Pain Med 2016;17:1676–85. 26755658 10.1093/pm/pnv079

[24] Darnall BD. Psychological treatment for patients with chronic pain. Washington D.C.: American Psychological Association, 2019.

[25] Davis MP. Twelve reasons for considering buprenorphine as a frontline analgesic in the management of pain. J Support Oncol 2012;10:209–19. 22809652 10.1016/j.suponc.2012.05.002

[26] De Ruddere L Craig KD. Understanding stigma and chronic pain: a-state-of-the-art review. PAIN 2016;157:1607–10. 26859821 10.1097/j.pain.0000000000000512

[27] Derefinko KJ García FIS Talley KM Bursac Z Johnson KC Murphy JG McDevitt-Murphy ME Andrasik F Sumrok DD. Adverse childhood experiences predict opioid relapse during treatment among rural adults. Addict Behav 2019;96:171–4. 31102882 10.1016/j.addbeh.2019.05.008

[28] Desmeules JA Cedraschi C Rapiti E Baumgartner E Finckh A Cohen P Dayer P Vischer T. Neurophysiologic evidence for a CENTRAL SENSITIZATION in patients with fibromyalgia. Arthritis Rheu 2003;48:1420–9. 10.1002/art.10893 12746916

[29] Doverty M Somogyi AA White JM Bochner F Beare CH Menelaou A Ling W. Methadone maintenance patients are cross-tolerant to the antinociceptive effects of morphine. PAIN 2001;93:155–63. 11427327 10.1016/S0304-3959(01)00306-2

[30] Doverty M White JM Somogyi AA Bochner F Ali R Ling W. Hyperalgesic responses in methadone maintenance patients. PAIN 2001;90:91–6. 11166974 10.1016/s0304-3959(00)00391-2

[31] Dudeney J Law EF Meyyappan A Palermo TM Rabbitts JA. Evaluating the psychometric properties of the widespread pain index and the symptom severity scale in youth with painful conditions. Can J Pain 2019;3:137–47. 32051925 10.1080/24740527.2019.1620097 PMC7015535

[32] Ellerbrock I Sandström A Tour J Kadetoff D Schalling M Jensen KB Kosek E. Polymorphisms of the μ‐opioid receptor gene influence cerebral pain processing in fibromyalgia. Eur J Pain 2021;25:398–414. 33064887 10.1002/ejp.1680 PMC7821103

[33] Elman I Borsook D. Common brain mechanisms of chronic pain and addiction. Neuron 2016;89:11–36. 26748087 10.1016/j.neuron.2015.11.027

[34] Finan PH Zautra AJ Davis MC Lemery-Chalfant K Covault J Tennen H. Genetic influences on the dynamics of pain and affect in fibromyalgia. Health Psychol 2010;29:134. 20230086 10.1037/a0018647 PMC3212833

[35] Fishbain DA Pulikal A. Does opioid tapering in chronic pain patients result in improved pain or same pain vs increased pain at taper completion? A structured evidence-based systematic review. Pain Med 2018;20:2179–97. 10.1093/pm/pny231 30597076

[36] Fitzcharles MA Cohen SP Clauw DJ Littlejohn G Usui C Hauser W. Nociplastic pain: towards an understanding of prevalent pain conditions. Lancet 2021;397:2098–110. 34062144 10.1016/S0140-6736(21)00392-5

[37] Fredheim O Moksnes K Borchgrevink P Kaasa S Dale O. Clinical pharmacology of methadone for pain. Acta Anaesthesiol Scand 2008;52:879–89. 18331375 10.1111/j.1399-6576.2008.01597.x

[38] George O Koob GF Vendruscolo LF. Negative reinforcement via motivational withdrawal is the driving force behind the transition to addiction. Psychopharmacology 2014;231:3911–17. 24923982 10.1007/s00213-014-3623-1 PMC8278497

[39] Gottrup H Juhl G Kristensen AD Lai R Chizh BA Brown J Bach FW Jensen TS. Chronic oral gabapentin reduces elements of CENTRAL SENSITIZATION in human experimental hyperalgesia. J Am Soc Anesth 2004;101:1400–8. 10.1097/00000542-200412000-00021 15564948

[40] Gudin J Fudin J. A narrative pharmacological review of buprenorphine: a unique opioid for the treatment of chronic pain. Pain Ther 2020;9:41–54. 31994020 10.1007/s40122-019-00143-6 PMC7203271

[41] Harris RE Clauw DJ Scott DJ McLean SA Gracely RH Zubieta J-K. Decreased central μ-opioid receptor availability in fibromyalgia. J Neurosci 2007;27:10000–6. 17855614 10.1523/JNEUROSCI.2849-07.2007 PMC6672650

[42] Harte S Clauw D Clauw A Scott R Moser S Brummett C. The 2011 fibromyalgia (FM) survey criteria are a surrogate measure of pain centralization: Abstract number: 2290. Arthritis Rheu 2015;67:2764–5.

[43] Harte SE Harris RE Clauw DJ. The neurobiology of CENTRAL SENSITIZATION. J Appl Biobehav Res 2018;23:e12137. 10.1111/jabr.12135 PMC6251410 30479469

[44] Hawkins JL Moore NJ Miley D Durham PL. Secondary traumatic stress increases expression of proteins implicated in peripheral and CENTRAL SENSITIZATION of trigeminal neurons. Brain Res 2018;1687:162–72. 29522721 10.1016/j.brainres.2018.03.003 PMC5882570

[45] Hays RD Morales LS. The RAND-36 measure of health-related quality of life. Ann Med 2001;33:350–7. 11491194 10.3109/07853890109002089

[46] Hooten WM Townsend CO Sletten CD Bruce BK Rome JD. Treatment outcomes after multidisciplinary pain rehabilitation with analgesic medication withdrawal for patients with fibromyalgia. Pain Med 2007;8:8–16. 17244099 10.1111/j.1526-4637.2007.00253.x

[47] Ibrahim C Rubin-Kahana DS Pushparaj A Musiol M Blumberger DM Daskalakis ZJ Zangen A Le Foll B. The insula: a brain stimulation target for the treatment of addiction. Front Pharmacol 2019;10:720. 31312138 10.3389/fphar.2019.00720 PMC6614510

[48] Imbierowicz K Egle UT. Childhood adversities in patients with fibromyalgia and somatoform pain disorder. Eur J Pain 2003;7:113–19. 12600792 10.1016/S1090-3801(02)00072-1

[49] International Association for the Study of Pain. IASP terminology. Available at: https://www.iasp-pain.org/terminology?navItemNumber=576#Centralsensitization. Accessed January 3, 2022.

[50] Janda AM As-Sanie S Rajala B Tsodikov A Moser SE Clauw DJ Brummett CM. Fibromyalgia survey criteria are associated with increased postoperative opioid consumption in women undergoing hysterectomy. Anesthesiology 2015;122:1103–11. 25768860 10.1097/ALN.0000000000000637

[51] Koob GF. Neurobiological substrates for the dark side of compulsivity in addiction. Neuropharmacology 2009;56:18–31. 18725236 10.1016/j.neuropharm.2008.07.043 PMC2637927

[52] Koob GF. Negative reinforcement in drug addiction: the darkness within. Curr Opin Neurobiol 2013;23:559–63. 23628232 10.1016/j.conb.2013.03.011

[53] Koob GF. Drug addiction: hyperkatifeia/negative reinforcement as a framework for medications development. Pharmacol Rev 2021;73:163–201. 33318153 10.1124/pharmrev.120.000083 PMC7770492

[54] Koob GF Buck CL Cohen A Edwards S Park PE Schlosburg JE Schmeichel B Vendruscolo LF Wade CL Whitfield TW George O. Addiction as a stress surfeit disorder. Neuropharmacology 2014;76:370–82. 23747571 10.1016/j.neuropharm.2013.05.024 PMC3830720

[55] Kouya PF Xu XJ. Buprenorphine reduces CENTRAL SENSITIZATION after repetitive C-fiber stimulation in rats. Neurosci Lett 2004;359:127–9. 15050727 10.1016/j.neulet.2004.02.007

[56] Kreutzwiser D Tawfic QA. Methadone for pain management: a pharmacotherapeutic review. CNS Drugs 2020;34:827–39. 32564328 10.1007/s40263-020-00743-3

[57] Lagisetty P Kehne AR Thomas J Yaganti A Slat S Patel S Macleod C Bicket MC Bohnert AS Gomirzaie G. Improving Access to Primary and Pain Care for Patients Taking Opioids for Chronic Pain in Michigan: Recommendations from an Expert Panel. Available at: https://ihpi.umich.edu/sites/default/files/2021-08/0240_Chronic-Pain-Brief_Lagisetty-final-08032021.pdf. August, 2021. Accessed June 12, 2022.

[58] Larson MJ Paasche‐Orlow M Cheng DM Lloyd‐Travaglini C Saitz R Samet JH. Persistent pain is associated with substance use after detoxification: a prospective cohort analysis. Addiction 2007;102:752–60. 17506152 10.1111/j.1360-0443.2007.01759.x

[59] Leza L Siria S López-Goñi JJ Fernandez-Montalvo J. Adverse childhood experiences (ACEs) and substance use disorder (SUD): a scoping review. Drug Alcohol Depen 2021:108563. 10.1016/j.drugalcdep.2021.108563 33561668

[60] Marion Lee M Sanford Silverman M Hans Hansen M Vikram Patel M Laxmaiah Manchikanti M. A comprehensive review of opioid-induced hyperalgesia. Pain Physician 2011;14:145–61. 21412369

[61] McKernan LC Johnson BN Crofford LJ Lumley MA Bruehl S Cheavens JS. Posttraumatic stress symptoms mediate the effects of trauma exposure on clinical indicators of CENTRAL SENSITIZATION in patients with chronic pain. Clin J Pain 2019;35:385–93. 30730446 10.1097/AJP.0000000000000689 PMC6450707

[62] Merrick MT Ford DC Haegerich TM Simon T. Adverse childhood experiences increase risk for prescription opioid misuse. J Prim Prev 2020;41:139–52. 31989435 10.1007/s10935-020-00578-0 PMC10976456

[63] Miettinen T Kautiainen H Mäntyselkä P Linton SJ Kalso E. Pain interference type and level guide the assessment process in chronic pain: categorizing pain patients entering tertiary pain treatment with the Brief Pain Inventory. PLoS One 2019;14:e0221437. 31430355 10.1371/journal.pone.0221437 PMC6701883

[64] Moorer P Suurmeijer TP Foets M Molenaar I. Psychometric properties of the RAND-36 among three chronic disease (multiple sclerosis, rheumatic diseases and COPD) in The Netherlands. Qual Life Res 2001;10:637–45. 11822796 10.1023/a:1013131617125

[65] Naqvi NH Bechara A. The hidden island of addiction: the insula. Trends Neurosci 2009;32:56–67. 18986715 10.1016/j.tins.2008.09.009 PMC3698860

[66] Neville SJ Clauw AD Moser SE Urquhart AG Clauw DJ Brummett CM Harte SE. Association between the 2011 fibromyalgia survey criteria and multisite pain sensitivity in knee osteoarthritis. Clin J Pain 2018;34:909–17. 29642237 10.1097/AJP.0000000000000619 PMC6110956

[67] Nicol A Arnold P Clauw D. Fibromyalgia-ness in persistent low back pain after lumbar spine surgery: a preliminary investigation. J Pain 2017;18:S55.

[68] Nijs J Leysen L Vanlauwe J Logghe T Ickmans K Polli A Malfliet A Coppieters I Huysmans E. Treatment of CENTRAL SENSITIZATION in patients with chronic pain: time for change?. Expert Opin Pharmaco 2019;20:1961–70. 10.1080/14656566.2019.1647166 31355689

[69] Oaks Z Stage A Middleton B Faraone SV Johnson B. Clinical utility of the cold pressor test: evaluation of pain patients and treatment of opioid-induced hyperalgesia and fibromyalgia with low dose naltrexone. Discov Med 2018;26:197–206. 30695679

[70] Olivieri P Solitar B Dubois M. Childhood risk factors for developing fibromyalgia. Open Access Rheumatol 2012;4:109–14. 27790019 10.2147/OARRR.S36086 PMC5045103

[71] Pierce J Hassett AL Brummett CM McAfee J Sieberg C Schrepf A Harte SE. Characterizing pain and generalized sensory sensitivity according to trauma history among patients with knee osteoarthritis. Ann Behav Med 2020;55:853–69. 10.1093/abm/kaaa105 PMC8382144 33377478

[72] Potvin S Marchand S. Pain facilitation and pain inhibition during conditioned pain modulation in fibromyalgia and in healthy controls. PAIN 2016;157:1704–10. 27045524 10.1097/j.pain.0000000000000573

[73] RAND Corporation. 36-Item Short Form Survey (SF‐36) Scoring Instructions. https://www.rand.org/health-care/surveys_tools/mos/36-item-short-form/scoring.html. Accessed June 12, 2022.

[74] Ren ZY Shi J Epstein DH Wang J Lu L. Abnormal pain response in pain-sensitive opiate addicts after prolonged abstinence predicts increased drug craving. Psychopharmacology 2009;204:423–9. 19172249 10.1007/s00213-009-1472-0 PMC3697848

[75] Reynolds M Mezey G Chapman M Wheeler M Drummond C Baldacchino A. Co-morbid post-traumatic stress disorder in a substance misusing clinical population. Drug Alcohol Depen 2005;77:251–8. 10.1016/j.drugalcdep.2004.08.017 15734225

[76] Ruscheweyh R Sandkühler J. Opioids and central sensitisation: II. Induction and reversal of hyperalgesia. Eur J Pain 2005;9:149–52. 15737805 10.1016/j.ejpain.2004.05.011

[77] Savage SR Kirsh KL Passik SD. Challenges in using opioids to treat pain in persons with substance use disorders. Addict Sci Clin Pract 2008;4:4–25. 18497713 10.1151/ascp08424 PMC2797112

[78] Shipley M. Chronic widespread pain and fibromyalgia syndrome. Medicine 2018;46:252–5.

[79] Smallwood RF Laird AR Ramage AE Parkinson AL Lewis J Clauw DJ Williams DA Schmidt-Wilcke T Farrell MJ Eickhoff SB Robin DA. Structural brain anomalies and chronic pain: a quantitative meta-analysis of gray matter volume. J Pain 2013;14:663–75. 23685185 10.1016/j.jpain.2013.03.001 PMC4827858

[80] Solak Ö Erdoğan M Ö Yıldız H Ulaşlı AM Yaman F Terzi ESA Ulu S Dündar Ü Solak M. Assessment of opioid receptor μ1 gene A118G polymorphism and its association with pain intensity in patients with fibromyalgia. Rheumatol Int 2014;34:1257–61. 24671502 10.1007/s00296-014-2995-1

[81] Speed TJ Parekh V Coe W Antoine D. Comorbid chronic pain and opioid use disorder: literature review and potential treatment innovations. Int Rev Psyhciatry 2018;30:136–46. 10.1080/09540261.2018.1514369 30398071

[82] Stein MD Conti MT Kenney S Anderson BJ Flori JN Risi MM Bailey GL. Adverse childhood experience effects on opioid use initiation, injection drug use, and overdose among persons with opioid use disorder. Drug Alcohol Depen 2017;179:325–9. 10.1016/j.drugalcdep.2017.07.007 PMC5599365 28841495

[83] Stumbo SP Yarborough BJH McCarty D Weisner C Green CA. Patient-reported pathways to opioid use disorders and pain-related barriers to treatment engagement. J Subst Abuse Treat Treatment 2017;73:47–54. 10.1016/j.jsat.2016.11.003 PMC5193128 28017184

[84] Trafton JA Oliva EM Horst DA Minkel JD Humphreys K. Treatment needs associated with pain in substance use disorder patients: implications for concurrent treatment. Drug Alcohol Depen 2004;73:23–31. 10.1016/j.drugalcdep.2003.08.007 14687956

[85] Trofimovitch D Baumrucker SJ. Pharmacology update: low-dose naltrexone as a possible nonopioid modality for some chronic, nonmalignant pain syndromes. Am J Hosp Palliat Care 2019;36:907–12. 30917675 10.1177/1049909119838974

[86] Vander Zee KI Sanderman R Heyink JW de Haes H. Psychometric qualities of the RAND 36-Item Health Survey 1.0: a multidimensional measure of general health status. Int J Behav Med 1996;3:104–22. 16250758 10.1207/s15327558ijbm0302_2

[87] VanderZee K Sanderman R Heyink J. A comparison of two multidimensional measures of health status: the nottingham health profile and the RAND 36-item health survey 1.0. Qual Life Res 1996;5:165–74. 8901380 10.1007/BF00435982

[88] Varinen A Kosunen E Mattila K Koskela T Sumanen M. The relationship between childhood adversities and fibromyalgia in the general population. J Psychosom Res 2017;99:137–42. 28712419 10.1016/j.jpsychores.2017.06.011

[89] Volkow ND Koob G. Brain disease model of addiction: why is it so controversial? Lancet Psychiat 2015;2:677–9. 10.1016/S2215-0366(15)00236-9 PMC4556943 26249284

[90] Wachholtz A Foster S Cheatle M. Psychophysiology of pain and opioid use: implications for managing pain in patients with an opioid use disorder. Drug Alcohol Depen 2015;146:1–6. 10.1016/j.drugalcdep.2014.10.023 PMC4272859 25468815

[91] Wachholtz A Gonzalez G Ziedonis D. Psycho-physiological response to pain among individuals with comorbid pain and opioid use disorder: implications for patients with prolonged abstinence. Am J Drug Alcohol Ab 2019;45:495–505. 10.1080/00952990.2019.1620260 PMC6684104 31246117

[92] Wanigasekera V Lee MC Rogers R Hu P Tracey I. Neural correlates of an injury-free model of CENTRAL SENSITIZATION induced by opioid withdrawal in humans. J Neurosci 2011;31:2835–42. 21414905 10.1523/JNEUROSCI.5412-10.2011 PMC3095083

[93] Weiss RD Potter JS Griffin ML McHugh RK Haller D Jacobs P Gardin J II Fischer D Rosen KD. Reasons for opioid use among patients with dependence on prescription opioids: the role of chronic pain. J Subst Abuse Treat 2014;47:140–5. 24814051 10.1016/j.jsat.2014.03.004 PMC4074437

[94] Wolfe F. Pain extent and diagnosis: development and validation of the regional pain scale in 12,799 patients with rheumatic disease. J Rheumatol 2003;30:369–78. 12563698

[95] Wolfe F. Fibromyalgianess. Artrit Rheum-Arthr 2009;6:715‐16. 10.1002/art.24553 19479689

[96] Wolfe F Clauw DJ Fitzcharles M-A Goldenberg DL Häuser W Katz RL Mease PJ Russell AS Russell IJ Walitt B. 2016 Revisions to the 2010/2011 fibromyalgia diagnostic criteria. Semin Arthritis Rheum 2016;46:319–29. 27916278 10.1016/j.semarthrit.2016.08.012

[97] Wolfe F Clauw DJ Fitzcharles M-A Goldenberg DL Häuser W Katz RS Mease P Russell AS Russell IJ Winfield JB. Fibromyalgia criteria and severity scales for clinical and epidemiological studies: a modification of the ACR Preliminary Diagnostic Criteria for Fibromyalgia. J Rheumatol 2011;38:1113–22. 21285161 10.3899/jrheum.100594

[98] Wolfe F Clauw DJ Fitzcharles MA Goldenberg DL Katz RS Mease P Russell AS Russell IJ Winfield JB Yunus MB. The American College of Rheumatology preliminary diagnostic criteria for fibromyalgia and measurement of symptom severity. Arthrit Care Res 2010;62:600–10. 10.1002/acr.20140 20461783

[99] Wolfe F Häuser W Hassett AL Katz RS Walitt BT. The development of fibromyalgia–I: examination of rates and predictors in patients with rheumatoid arthritis (RA). PAIN 2011;152:291–9. 20961687 10.1016/j.pain.2010.09.027

[100] Woolf CJ. Pain amplification—a perspective on the how, why, when, and where of CENTRAL SENSITIZATION. J Appl Biobehav Res 2018;23:e12124.

[101] You DS Meagher MW. Childhood adversity and pain sensitization. Psychosom Med 2016;78:1084–93. 27755280 10.1097/PSY.0000000000000399

[102] Zhang Y Shao G Zhang W Li S Niu J Hu D Yang M Ji X. Gabapentin inhibits CENTRAL SENSITIZATION during migraine. Neural Regen Res 2013;8:3003. 25206620 10.3969/j.issn.1673-5374.2013.32.003 PMC4146212

